# The lateral mobility of cell adhesion molecules is highly restricted at septate junctions in *Drosophila*

**DOI:** 10.1186/1471-2121-9-38

**Published:** 2008-07-18

**Authors:** Monique Laval, Christophe Bel, Catherine Faivre-Sarrailh

**Affiliations:** 1 Centre de Recherche en Neurobiologie et Neurophysiologie de Marseille, UMR 6231 CNRS, Marseille, France; 2 Université de la Méditerranée Aix-Marseille II, Faculté de Médecine Nord, 13916 Marseille, France

## Abstract

**Background:**

A complex of three cell adhesion molecules (CAMs) Neurexin IV(Nrx IV), Contactin (Cont) and Neuroglian (Nrg) is implicated in the formation of septate junctions between epithelial cells in *Drosophila*. These CAMs are interdependent for their localization at septate junctions and e.g. null mutation of *nrx IV *or *cont *induces the mislocalization of Nrg to the baso-lateral membrane. These mutations also result in ultrastructural alteration of the strands of septate junctions and breakdown of the paracellular barrier. Varicose (Vari) and Coracle (Cora), that both interact with the cytoplasmic tail of Nrx IV, are scaffolding molecules required for the formation of septate junctions.

**Results:**

We conducted photobleaching experiments on whole living *Drosophila *embryos to analyze the membrane mobility of CAMs at septate junctions between epithelial cells. We show that GFP-tagged Nrg and Nrx IV molecules exhibit very stable association with septate junctions in wild-type embryos. Nrg-GFP is mislocalized to the baso-lateral membrane in *nrx IV *or *cont *null mutant embryos, and displays increased mobile fraction. Similarly, Nrx IV-GFP becomes distributed to the baso-lateral membrane in null mutants of *vari *and *cora*, and its mobile fraction is strongly increased. The loss of Vari, a MAGUK protein that interacts with the cytoplasmic tail of Nrx IV, has a stronger effect than the null mutation of *nrx IV *on the lateral mobility of Nrg-GFP.

**Conclusion:**

The strands of septate junctions display a stable behavior *in vivo *that may be correlated with their role of paracellular barrier. The membrane mobility of CAMs is strongly limited when they take part to the multimolecular complex forming septate junctions. This restricted lateral diffusion of CAMs depends on both adhesive interactions and clustering by scaffolding molecules. The lateral mobility of CAMs is strongly increased in embryos presenting alteration of septate junctions. The stronger effect of *vari *by comparison with *nrx IV *null mutation supports the hypothesis that this scaffolding molecule may cross-link different types of CAMs and play a crucial role in stabilizing the strands of septate junctions.

## Background

In insects, septate junctions localize to the apico-lateral domain of epithelial cells underneath the zonula adherens and play the role of the vertebrate tight junctions by preventing paracellular diffusion [1]. Septate junctions are also found between the glial cells that isolate the brain and peripheral nerves and are required for blood-nerve barrier formation in *Drosophila *[2]. The machinery providing axonal insulation has been conserved for a part during evolution since, in vertebrate myelinated axons, septate-like junctions attach the terminal myelin loops to the axonal membrane at paranodes. Both the ultrastructural feature and the molecular composition of *Drosophila *septate junctions and vertebrate paranodal junctions are highly conserved [3,4]. Septate junctions are formed by strands of regularly spaced inter-membrane particles. A complex of CAMs including Nrx IV, Cont and Nrg is critically involved in the organization of septate junctions in *Drosophila *whereas the homologous molecules, Caspr/Paranodin, Contactin and Neurofascin-155 are required for the formation of paranodal junctions in vertebrates [5].

Nrx IV is a transmembrane molecule with a multimodular ectodomain including several laminin-G and EGF-like domains. Nrx IV interacts in cis with Cont, a glypiated CAM of the Ig superfamily (Ig-CAM), which only displays heterophilic binding activity [6,7]. Nrg is a homophilic transmembrane Ig-CAM of the L1 family [8,9] that associates with Cont and Nrx IV at septate junctions. The cytoplasmic tail of Nrx IV contains a binding site for Coracle (Cora), a member of the Four-point-one, Ezrin, Radixin, Moesin (FERM) family [10]. It also includes a C-ter PDZ-binding motif interacting with the membrane associated guanylate kinase (MAGUK) Varicose (Vari) [11].

In *Drosophila*, null mutation of each of the *nrx IV*, *cont *or *nrg *genes induces embryonic lethality with breakdown of the trans-epithelial and blood-nerve barriers and alteration of the septate junctions [6,12,13]. In epithelial cells of *nrx IV *null mutant, Nrg is misdistributed to the baso-lateral membrane and Cont is diffusely distributed in the cytoplasm indicating that Nrx IV is required for the proper membrane expression of Cont. Reciprocally in *cont *null mutant, Nrx IV and Nrg are mislocalized to the baso-lateral membrane. Null mutation for *cora *or *vari *coding for scaffolding molecules of septate junctions cause barrier defects and mislocalization of Nrx IV. In addition, the PDZ-containing proteins Discs large (Dlg) and Scribble localized at septate junctions, also control the establishment of epithelial cell polarity [18]. Several other membrane molecules have been identified as critical components of the fly septate junctions, including Gliotactin [14], Lachesin [15] and the claudin-related molecules Megatrachea [16] and Sinuous [17]. The loss of one of the septate junction membrane components induces alteration of the trans-epithelial barrier and mislocalization of the Nrx IV complex at the baso-lateral region although the network of interactions bridging together these molecules is still unknown.

An interesting question is whether the strands of septate junctions display either dynamic characteristics or stable behavior to achieve their role of barrier. *Drosophila *offers a model system to investigate the membrane mobility of CAMs when recruited into highly ordered multimolecular complexes at septate junctions of epithelial cells and when these complexes are disrupted in different genetic backgrounds. We addressed this question using Fluorescence Recovery After Photobleaching (FRAP) analysis in live embryos.

## Results and discussion

### The lateral mobility of Nrg is restricted at septate junctions of epithelial cells in whole living embryos

We used a *Nrg-GFP *Flytrap line where a chimera between Nrg and GFP is expressed from the endogenous Nrg promoter as described in Morin et al. [19] and reflects the tissue and subcellular distribution of the endogenous protein [20,21]. GFP is inserted in the cytoplasmic tail close to the C-ter of Nrg. The homozygous line is viable and fertile without any apparent phenotype.

**Figure 1:**
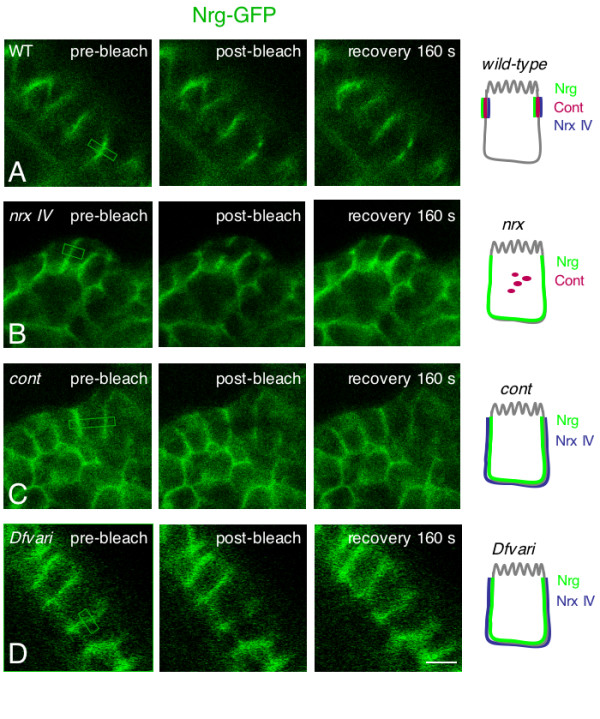
**FRAP of GFP-tagged Nrg in epithelial cells of live embryos**. Side views of epithelial cells in wild-type (A), *nrx IV *(B), *cont *(C) and Df*vari *(D) embryos at stage15. Pre-bleach images are shown on the left. The sections of cell interface that were bleached are indicated with boxes. Images on the right show the recovery of fluorescence 160 seconds after bleaching. Bar: 5 μm. Schematic representation of the distribution of septate junction components, Nrg, Nrx IV and Cont in each genotype.

**Figure 2:**
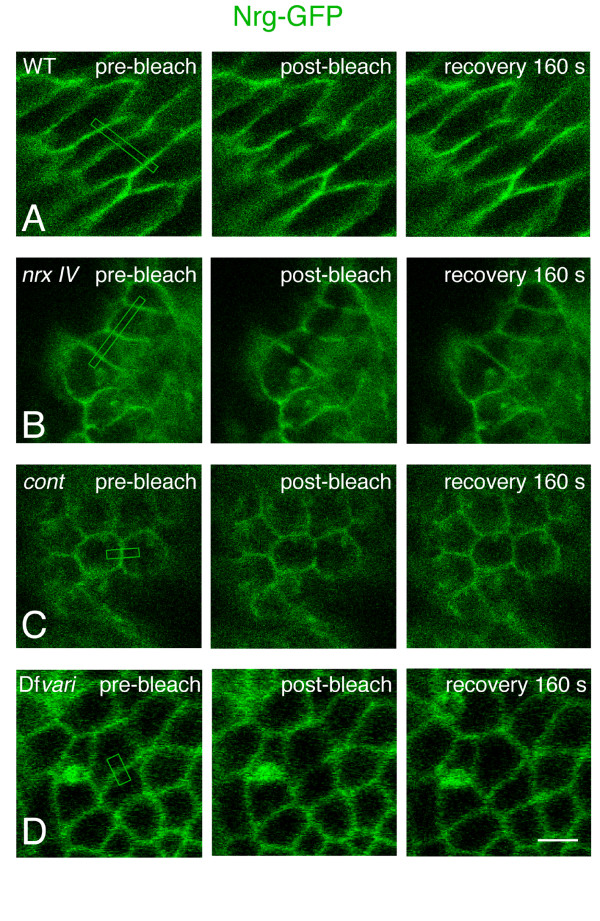
**FRAP of Nrg-GFP in embryonic epithelial cells**. Face-on views of epithelial cells in wild-type (A), *nrx IV *(B), *cont *(C) and Df*vari *(D) embryos at stage15. Pre-bleach images are shown on the left. The sections of cell interface that were bleached are indicated with boxes. Images on the right show the recovery of fluorescence 160 seconds after bleaching. Bar: 5 μm.

**Figure 3:**
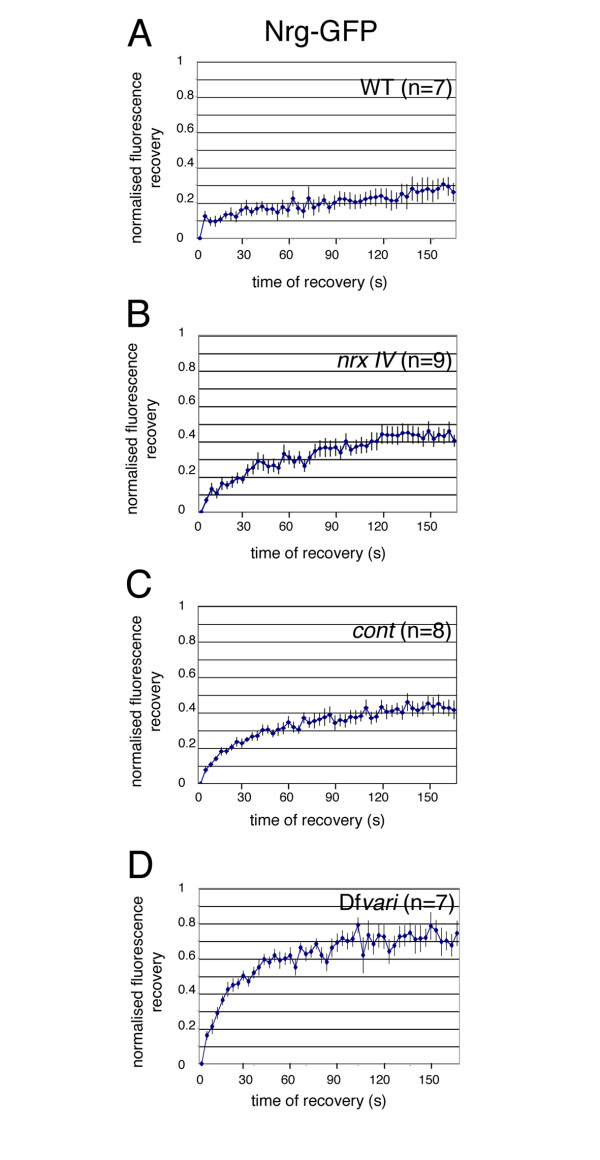
**Kinetics of Nrg-GFP fluorescence recovery**. Photobleaching in the horizontal plane of epithelial cells from wild-type (A), *nrx IV *(B) *cont *(C) and Df*vari *(D) embryos. Data are normalized to yield an intensity of zero immediately after photobleaching and represents the average of 7–9 individual traces (mean ± SEM). The traces were obtained using FRAP sequences from multiple embryos [wild-type (n = 3), *nrx *(n = 5), *cont *(n = 3) and *vari *(n = 2)]. Statistical analysis using ANOVA indicates that *nrx IV *and *cont *mutations significantly (P < 0.05) increased the mobile fraction of Nrg-GFP as compared with wild-type embryos. The mobile fraction of Nrg-GFP in Df*vari *embryos is significantly (P < 0.01) increased by comparison with wild-type, *nrx IV *and *cont *embryos.

We analyzed the lateral mobility of Nrg using FRAP in whole living embryos at stage 15. At this stage, Nrg-GFP expression is restricted to the apico-lateral domain in epithelial cells indicating it is recruited at septate junctions. A photobleach was applied in areas of septate junctions and the fluorescence recovery was monitored in the bleached spot. These experiments were usually not extended for longer time than 160 s because of extensive epidermis movements. Photobleaching of a 3–5 μm^2 ^area was performed either in the apico-lateral (Fig. 1) or horizontal (Fig. 2) orientation. In either orientation, the recovery of fluorescence in the bleached area was low in wild-type embryos, and the mobile fraction of Nrg-GFP estimated to 25 % within 160 s (Fig. 3A). We did not observe any difference between the ventral and dorsal epidermis. We also examined the long-term recovery of Nrg-GFP after photobleaching and only a partial recovery (39 %) occurred even after 40 min [see Additional file 1]. Thus, our data indicates that Nrg-GFP is strongly stabilized at the apico-lateral membrane by forming an adhesion complex at septate junctions of wild-type embryos. As a matter of comparison, a similar stability was reported for the structural components of the adherens junctions, E-cadherin, α-catenin and Armadillo as analyzed by FRAP in live *Drosophila *embryos [22].

### The lateral mobility of Nrg strongly increases in *nrx IV *and *cont *mutant embryos

In *nrx IV *null mutant embryos, Nrg-GFP mislocalized to the baso-lateral membrane of epithelial cells and its mobile fraction strongly increased (44 % within 160 s) by comparison with wild-type embryos (Fig. 1B, 2B and 3B). The high mobility of Nrg-GFP in *nrx IV *mutant embryos may be correlated with disruption of the septate junction tripartite complex. It must be noted that Cont is diffusely distributed in the cytoplasm of epithelial cells in *nrx IV *mutants [6] implicating that both Nrx IV and Cont are lacking at the epithelial cell membrane. The loss of *nrx IV *is accompanied by an almost complete lack of junctional strands as analyzed by electron microscopy [12].

**Figure 4:**
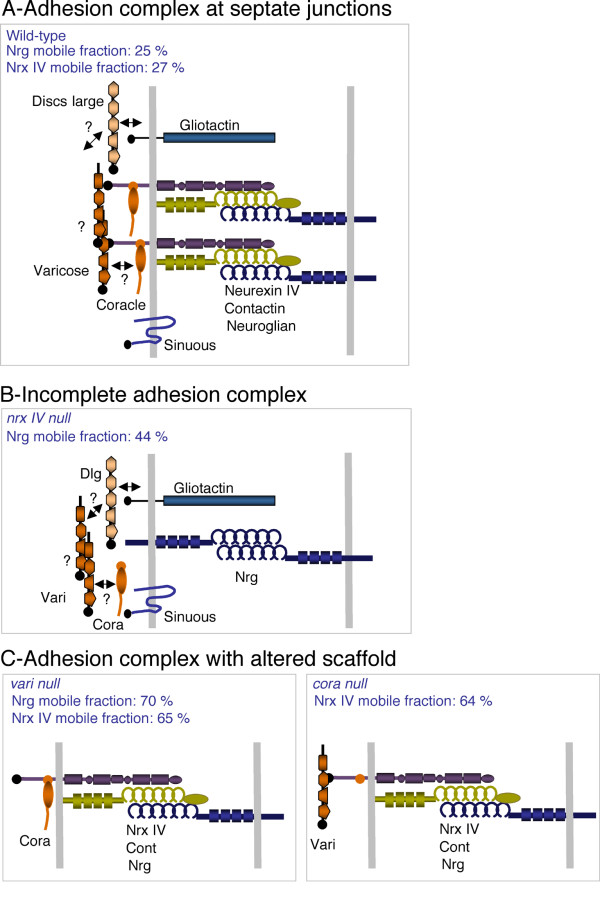
**Organization of adhesion complexes at epithelial cell contacts in the wild-type and mutant embryos**. (A) Molecular organization of septate junctions in wild-type embryos. NrxIV forms a tripartite complex with Cont and Nrg. A highly ordered complex may be generated due to the binding of Nrx IV with the scaffolding molecules Vari and Cora. Several additional components including Gliotactin, Sinuous and Dlg are required for the formation of septate junctions, but the network of interactions mediating bridging with the Nrx IV complex is largely unknown. Vari, which displays a multimodular structure, may homo-multimerize and mediate linkage with Cora, Dlg or Sinuous. FRAP analysis indicates that the mobile fraction of Nrg and Nrx IV is low. (B) In *nrx IV *mutant embryos, the tripartite complex is missing, Cont is not targeted to the cell membrane and Nrg may bind homophilically. The mobile fraction of Nrg is increased to 45 % indicating rapid exchange between bound and unbound molecules. However, the presence of Vari and Cora may limit for a part the lateral mobility of Nrg by cross-linking with other components of septate junctions. (C) In *cora *or *vari *null mutant embryos, the tripartite complex between Nrx IV, Cont and Nrg may still occur, but the network of interactions mediated by the scaffolding molecules is disrupted. The mobile fractions of Nrx IV and Nrg are increased up to 64–70 %.

Similarly, in *cont *mutants, Nrg-GFP was expressed at the baso-lateral membrane of epithelial cells and the mobile fraction of Nrg-GFP (40 % within the time of 160 s) strongly increased when compared with wild type embryos (Fig. 1C, 2C and 3C). In *cont *or *nrx IV *mutant embryos, Nrg does not take part into the tripartite adhesion complex of septate junctions but may still be engaged in homophilic binding between two neighboring epithelial cells [23,24]. Under these last conditions, exchange between free and bound Nrg-GFP receptors may occur rapidly allowing lateral diffusion along the plasma membrane (Fig. 4B).

### The restricted mobility of Nrg and Nrx IV at septate junctions depends on the scaffolding components Vari and Cora

**Figure 5:**
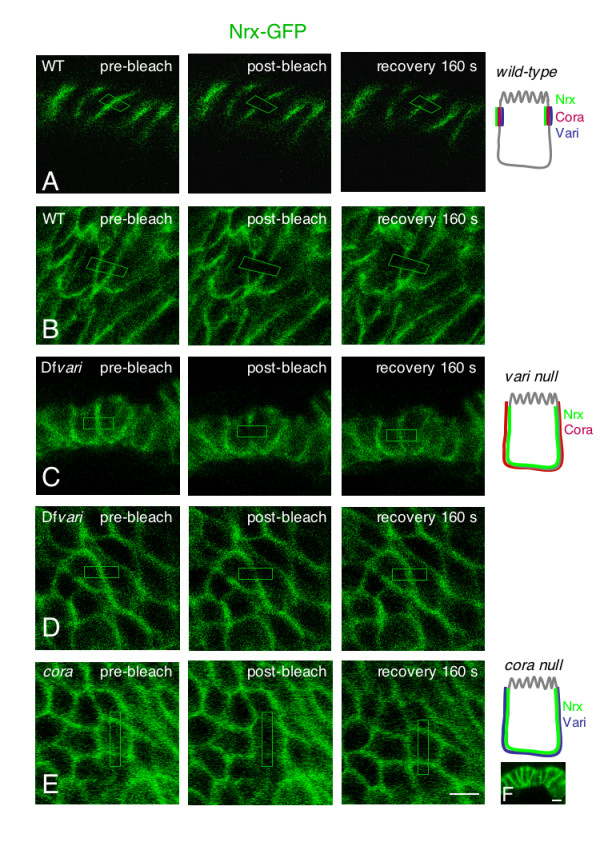
**FRAP of Nrx IV-GFP in embryonic epithelial cells**. Side (A) and face-on (B) views of wild-type embryos, side (C) and face-on (D) views of Df*vari *embryos at stage15. Face-on (E) and side (F) views of *cora *mutant embryos. Pre-bleach images are shown on the left. The bleached areas are indicated with boxes. Images on the right show the recovery of fluorescence 160 seconds after bleaching. Bar: 5 μm in A-E and F. Schematic representation of the distribution of septate junction components, Nrx IV, Vari and Cora in each genotype.

**Figure 6:**
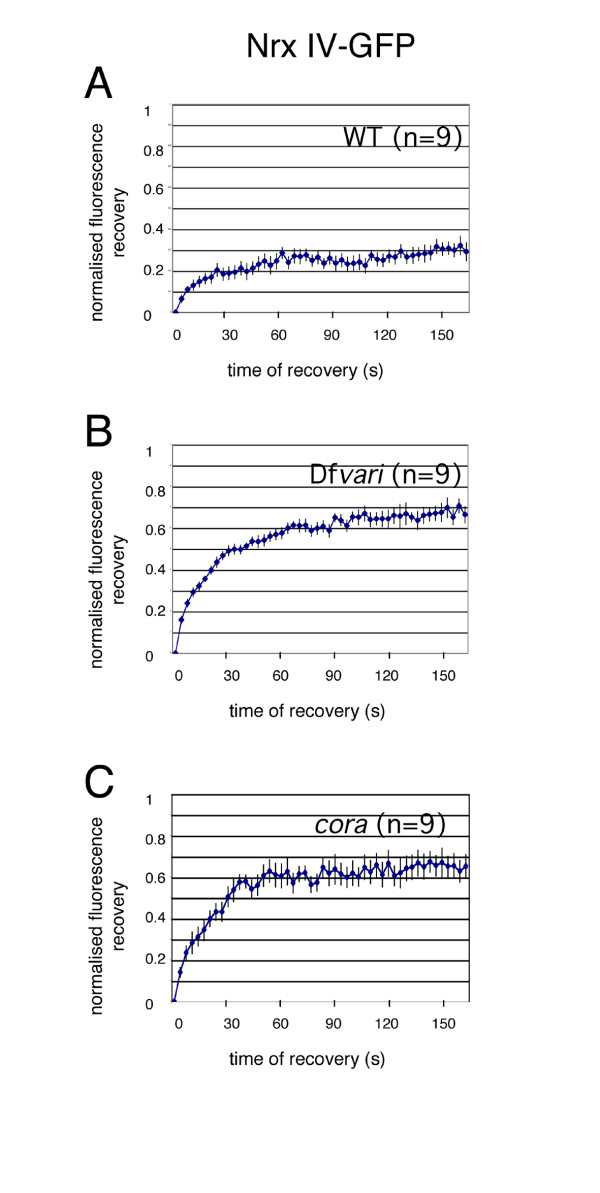
**Kinetics of the fluorescence recovery of Nrx IV-GFP**. Photobleaching in the horizontal plane of epithelial cells from wild-type (A), Df*vari *(B) and *cora *(C) embryos. Data are normalized to yield an intensity of zero immediately after photobleaching and represents the average of 9 individual traces (mean ± SEM). The traces were obtained using FRAP sequences from multiple embryos [wild-type (n = 3), *cor *(N = 4) and *vari *(N = 2)]. Statistical analysis using ANOVA indicates that the loss of Vari or Cora significantly (P < 0.01) increased the mobile fraction of Nrx IV-GFP as compared with wild-type embryos.

We used a Nrx IV-GFP Flytrap line where GFP is inserted into the first intron between the N-ter signal sequence and the discoïdin domain [25]. This line is homozygous viable and fertile and Nrx IV-GFP expression is restricted to the apico-lateral domain in epithelial cells of stage 12–15 embryos indicating it is recruited at septate junctions as the endogenous protein (Fig. 5A). We performed FRAP analysis on wild-type embryos at stage 15 and determined that the mobile fraction of Nrx IV-GFP was 27 % within 160 s in septate junctions of epithelial cells (Fig. 6A).

Next, we analyzed the mobility of both Nrg-GFP and Nrx IV-GFP in a deficiency (Df*vari*) for *vari*, which encodes a MAGUK that binds the cytoplasmic tail of Nrx IV [11]. As shown in Fig. 1D and 5C, the loss of *vari *induces the mislocalization of both Nrg-GFP and Nrx IV-GFP at the baso-lateral membrane. In this deficiency, the recovery fraction of Nrg-GFP after photobleaching is very high (70 % within the time of 160 s) (Fig. 1D, 2D and 3D). Similarly, the mobile fraction of Nrx IV-GFP (65 % within the time of 160 s) strongly increased by comparison with wild-type embryos (Fig. 5C–D and 6B). The MAGUK protein Vari that contains L27, PDZ, SH3, HOOK, and GUK modules may act as a scaffolding molecule at septate junctions. The PDZ domain of Vari binds Nrx IV and its HOOK domain may be implicated in homo-multimerization or anchoring Dlg, another MAGUK associated with septate junctions [26] and interacting with Gliotactin [14]. Indeed, mutations in the HOOK domain impair the accumulation of Vari at septate junctions [11]. Therefore Vari may support oligomerization of transmembrane components of the septate junction complex or induce linkage with submembrane elements (Fig. 4A).

It must be noted that the mobile fraction of Nrg-GFP is significantly increased in Df*vari *embryos by comparison with *nrx IV *null mutant embryos (70 % instead of 45 %). This is in accordance with genetic evidences indicating that Vari may display functions beyond interacting with Nrx IV and bridge together several elements of the septate junctions. As a matter of the fact, *vari *mutations can strongly enhance the phenotype caused by mutations of the claudin *sinuous*, whereas *nrx IV *mutations do not [17].

Finally, the mobility of Nrx IV-GFP was analyzed in a null allele for *cora *(Fig. 5E). Nrx IV-GFP localized at the baso-lateral membrane in the mutant embryos (Fig. 5F). As observed in Df*vari *embryos, the mobile fraction of Nrx IV-GFP (64 % within the time of 160 s) strongly increased by comparison with wild-type embryos (Fig. 6C). The FERM domain of Cora is required for the recruitment of Nrx IV at septate junctions [10]. However, how Cora would generate a scaffold is unknown since it does not contain an actin-spectrin binding domain like its related vertebrate homologue 4.1 adaptors. Our data suggest that Cora may participate to the septate junction scaffold beyond interacting with Nrx IV since its mutation has a similar effect as the loss of Vari.

### Lateral mobility of Caspr/Paranodin in transfected cells

**Figure 7:**
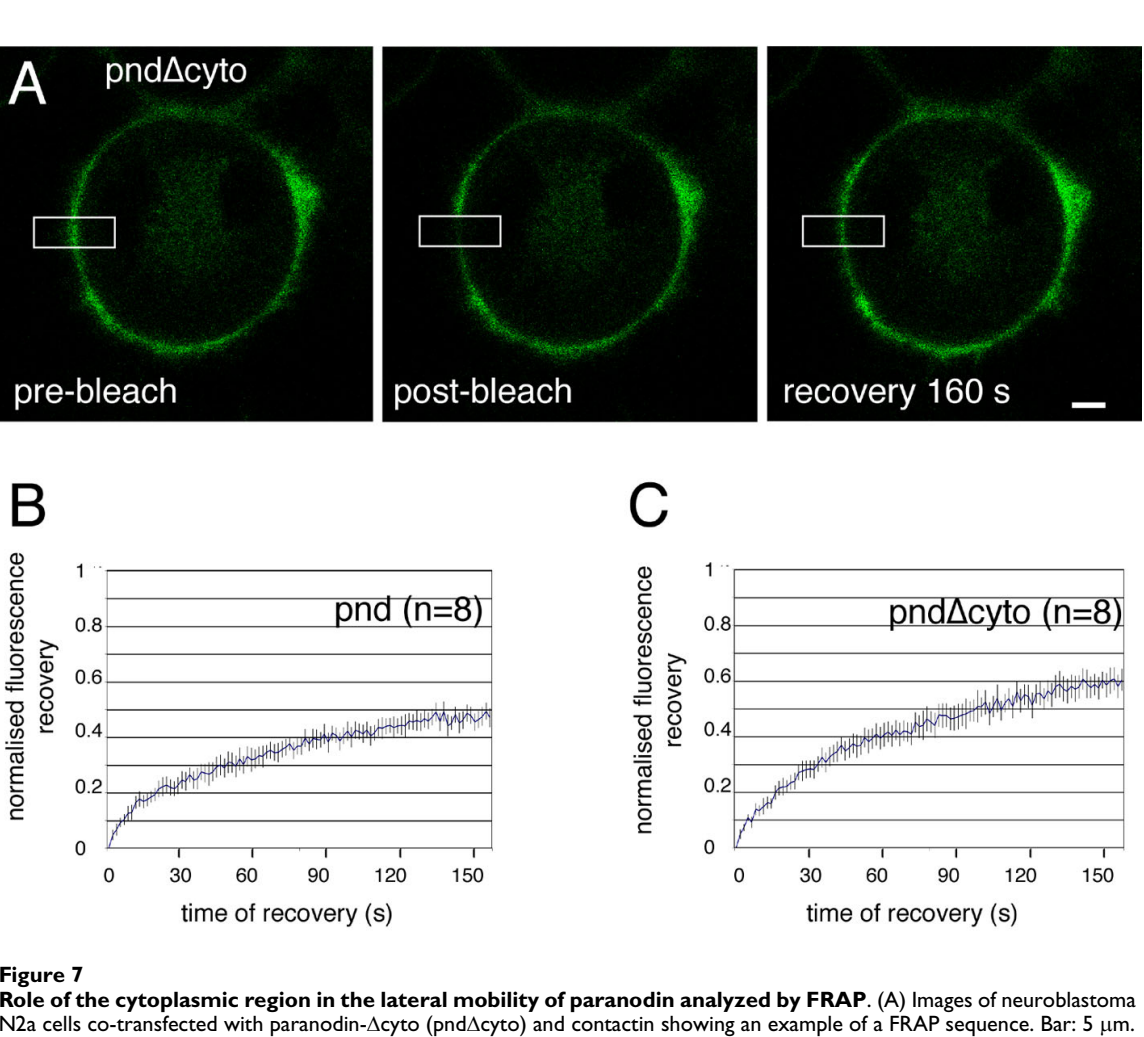
**Role of the cytoplasmic region in the lateral mobility of paranodin analyzed by FRAP**. (A) Images of neuroblastoma N2a cells co-transfected with paranodin-Δcyto (pndΔcyto) and contactin showing an example of a FRAP sequence. Bar: 5 μm. Kinetics of fluorescence recovery of GFP-tagged paranodin (B) and paranodinΔcyto (C). Data are normalized to yield an intensity of zero immediately after photobleaching and represents the average of 8 individual traces (mean ± SEM). Deletion of the cytoplasmic tail of paranodin significantly (P < 0.05) increased the mobile fraction of paranodin-GFP as assessed using ANOVA.

Next, we compared the dynamics of the Nrx IV CAM when anchored or not at septate junctions in live embryos with the mobility of its vertebrate orthologue Caspr/Paranodin at the membrane of transfected cells without any junctional complex or intercellular contacts. We examined the lateral mobility of Paranodin fused with GFP at the C-ter in neurobastoma N2a cells. Paranodin-GFP was co-transfected together with Contactin, which is required for its cell surface expression in vertebrate cells [27]. In this context, cell-cell contacts do not induce the formation of septate-like junctions as observed at the level of axo-glial paranodal junctions. FRAP experiments were performed by bleaching a section of cell membrane (Fig. 7A). The mean mobile fraction of Paranodin-GFP was 47 % within the time of 160 s (Fig. 7B).

We evaluated the role of the cytoplasmic region of Paranodin in its lateral mobility at the cell membrane. We analyzed the mobility of a mutant form, ParanodinΔcyto, with deletion of the cytoplasmic tail. The length of the C-ter of transmembrane proteins has been shown to influence their lateral mobility [28] since a minimal length should be required for the restriction by dynamic barriers. In the Paranodin-GFP constructs, GFP (265 aa) is fused at the C-ter and is expected to minimize the size effect of the cytoplasmic tail deletion (73 aa). This deletion mutant displayed a mobile fraction of 60 % (Fig. 7C), which is significantly enhanced by comparison with full-length Paranodin. Paranodin contains a juxta-membrane motif for the binding of 4.1 molecules, which are adaptors for linkage with the actin-spectrin cytoskeleton. Paranodin interacts with 4.1B at the paranodal junctions [29] similarly to Nrx IV that binds Cora in *Drosophila *septate junctions [10]. However, in contrast to Nrx IV, Paranodin does not contain a C-ter PDZ-binding domain. When co-transfected in Cos cells, Paranodin forming a complex with Contactin interacts with the endogenously expressed 4.1 molecule Schwannomin and deletion of the 4.1-binding motif prevents this interaction [30]. Thus, the association of the cytoplasmic tail of Paranodin with a 4.1 scaffolding molecule may reduce its lateral mobility.

## Conclusion

We report here the first *in vivo *measurements of the dynamics of septate junctions, which display similar stability as adherens junctions in Drosophila epithelial cells. Septate junctions form a barrier to trans-epithelial diffusion but may also act as a fence within the plane of the plasma membrane to prevent diffusion of membrane-bound proteins between the apical and basolateral membrane domains. However, septate junctions that are fully assembled at late stages of the embryogenesis do not play a critical role in the maintenance of membrane asymmetry in fly epithelial cells although they could play a redundant function together with adherens junctions [18]. In vertebrates, the septate-like junctions at paranodes play such a role of fence by limiting the lateral diffusion of the voltage-gated sodium and potassium channels along the axon [31]. Our data indicating that membrane proteins are stably anchored at the septate junctions are compatible with such a function of lateral barrier.

Our *in vivo *FRAP analyses indicate that the Nrg and Nrx IV molecules inserted in the strands of septate junctions are quite stable likely in terms of the arrangement of CAMs within strands and behavior of the strands. As observed for CAMs within septate junctions, claudin molecules are not mobile within paired strands as examined in artificially induced tight junction-like structures within apposing membranes of fibroblasts transfected with GFP-claudin-1 [32]. However, the network of claudin strands show a very dynamic behavior although this observation is not necessarily representative of tight junction strands in epithelial cells, because of the lack of ability of the GFP-claudin-1 to bind PDZ domain-containing proteins such as ZO-1, and then, to be cross-linked with the underlying cytoskeleton.

The restricted mobility of CAMs at septate junctions depends on both adhesive interactions and clustering by scaffolding molecules. The membrane mobility of Nrg-GFP and Nrx IV-GFP is strongly increased in mutant embryos presenting alteration of septate junctions. An important distinction between the effects of the loss of cell membrane and scaffolding components has been revealed by dynamic analysis. Mutations of septate junction CAMs significantly increase the mobility of the transmembrane components, but mutations in scaffolding elements have an even greater effect. These data underline the major role of scaffolding molecules in stabilizing highly ordered adhesion complexes at septate junctions.

## Methods

### Drosophila stocks

Stocks were obtained from the Bloomington Drosophila Stock Center and published sources *cont*^*ex*956 ^[6], *nrx IV*^4304 ^[12] and *cora*^1 ^[33]alleles have been described previously. The deficiency Df*vari *(w^1118^; Df(2L)Exel7079, P+PBac{XP5.WH5}Exel7079/CyO) was used as a null mutant for *vari *[11]. The Flytrap lines Nrg-GFP (G00305) [19] and Nrx IV-GFP (CA06597) [25] obtained from the FlyTrap project contain the GFP exon inserted in the *nrg *and *nrx IV *genes, respectively. New mutant lines were generated with the following phenotypes: *nrg-GFP; cont*^*ex*956^/*TM3, nrg-GFP; nrx IV*^4304^/*TM3, nrg-GFP; *Df*vari/CyO; cora*^1^/*CyO; nrx IV-GFP*, Df*vari/CyO; nrx IV-GFP*.

### Preparation of embryos

Embryos were collected on yeast agar plates and aged to stage 15 (12 h–13 h 30 at 25°C). The embryos were dechorionated for 2 min with bleach (6% chlore), rinsed with water, blotted dry with paper towels, gently picked up with double-sided tape and placed in a Petri dish and covered with water for FRAP analysis.

### Transfected cells and Caspr/Paranodin constructs

The DNA construct pRc-CMV/F3 encodes the full-length sequence of F3/Contactin, Paranodin-GFP encodes the full-length sequence and Paranodin-Δcyto encodes the extracellular and transmembrane domains of Caspr/Paranodin (aa1-1297) fused with GFP in pEGFP-N1 [34]. Neuroblastoma N2a cells plated on coverslips were grown in DMEM containing 10% FCS and transiently transfected using jet PEI (Ozyme, Saint Quentin Yvelines, France). The coverslips were placed in a Petri dish in a temperature controlled chamber at 37°C for FRAP analysis, 48-h after transfection.

### FRAP analysis

The FRAP analysis was performed on a Leica (Wetzlar, Germany) TCS SP2 laser scanning microscope equipped with 1.2 NA 63× water immersion objective. Imaging of GFP was performed using the 488-nm beam of an argon laser at 7% laser power, with the electronic zoom at 5×, scan speed of 400 Hz, box size 512 × 512 pixels. Images (two averages) were acquired with an open pinhole. These conditions were found to give minimal photobleaching over the observed time. The ROI for bleaching (3–5 μm^2^) was delineated and the FRAP sequence was started: 3–6 reference images were acquired first, then the sample was bleached by the laser at 100 % for 3 s (2 bleach scans), and fluorescence recovery was recorded for 160 s using the Leica FRAP software. Acquisition of z-stacks before and after bleaching indicates that the bleached spot can be detected over 2.8 μm in the z-axis [see Additional file 2]. Thus, even in case of epidermal movements in the z-axis, images of the recovery sequence are acquired within the bleached volume. In addition, all the images of the FRAP sequences were checked by eye to correct the ROI position for epidermal movements in xy. To create the recovery curves, the background-corrected fluorescence intensities were transformed into a 0–1 scale and plotted using Microsoft Excel. The individual traces were fitted using SigmaPlot through the data of the function I_m_*(1-exp(-t/τ)) to calculate the mobile fraction I_m_, and the recovery time τ.

Multiple embryos (2–5) for each genotype were analyzed. FRAP experiments were conducted in the same sample on heterozygous and homozygous embryos for each of the *nrx*, *cont*, *cor*, and *vari *mutations. Similar kinetics of fluorescence recovery were obtained for Nrg-GFP or Nrx IV-GFP in wild-type and heterozygous embryos.

## Authors' contributions

ML participated in the design of the study, carried out the fly genetic crossing, participated to the FRAP analysis and helped to draft the manuscript. CB carried out the FRAP analysis in neuroblastoma cells and performed the statistical analysis. CF–S conceived the study, performed FRAP analysis on Drosophila embryos and drafted the manuscript. All authors read and approved the final manuscript.

## Supplementary Material

Additional file 1 Long term recovery of Nrg-GFP after photobleaching in epithelial cells of live embryos. Face-on views of epithelial cells in wild-type embryos. Pre-bleach image with the bleached area indicated with box. Note the partial recovery of fluorescence even until 41 min after bleaching. Bar: 5 μm. Click here for file

Additional file 2 Bleaching of Nrx IV-GFP along the z-axis in epithelial cells of live embryos. Confocal xy- and z-sections (stacks of 8 sections, 400 nm step) of epithelial cells in wild-type embryos. Pre-bleach and post-bleach images with the bleached area indicated with box. Quantification of fluorescence intensities into the bleached area of confocal sections. Bar: 5 μm. Click here for file
