# Supplementary figures and images for: The lateral mobility of cell adhesion molecules is highly restricted at septate junctions in Drosophila
Source: BMC Cell Biol. 2008 Jul 18;9:38. doi: 10.1186/1471-2121-9-38 (PMC2500017; doi:10.1186/1471-2121-9-38)

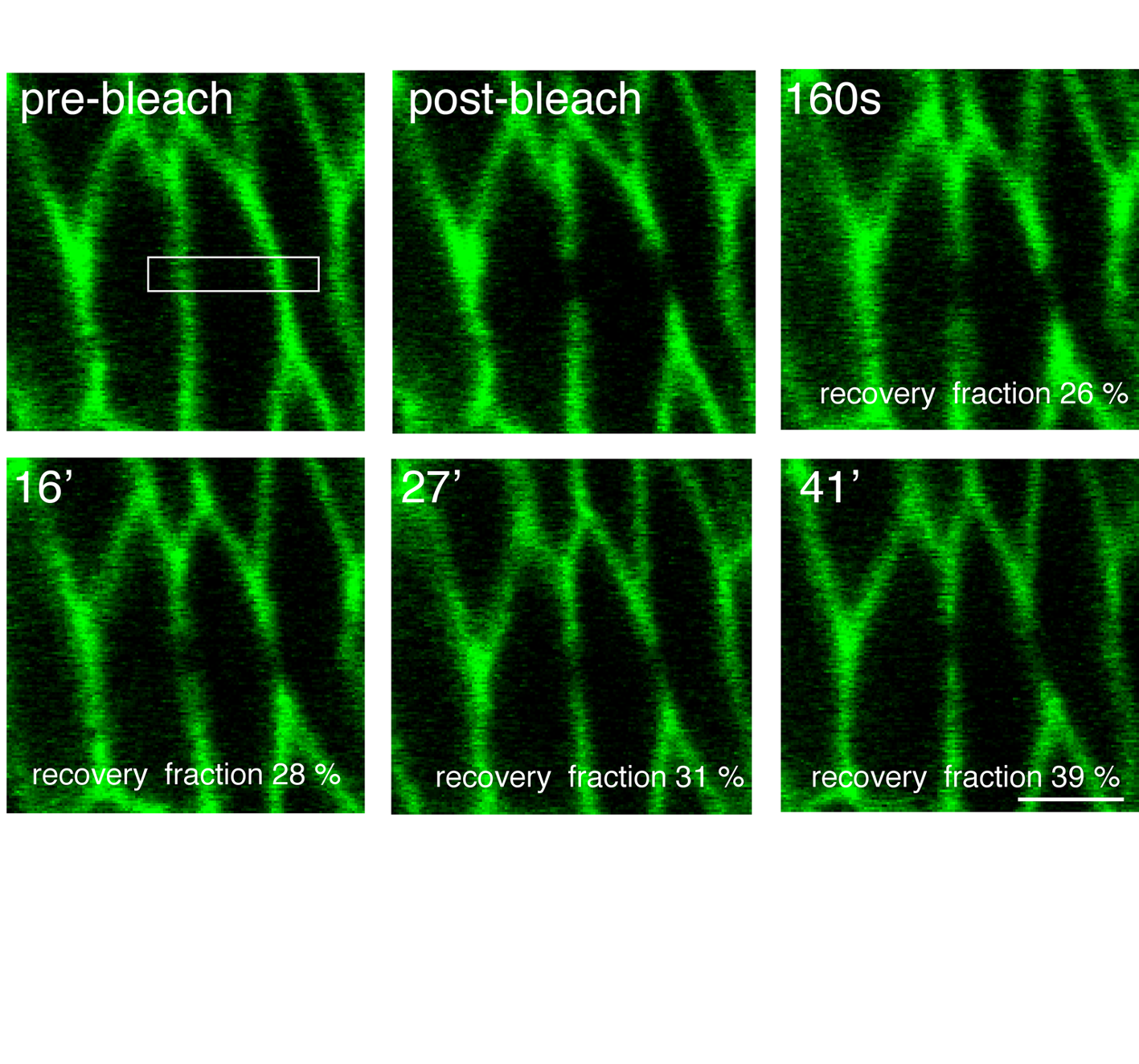

Supplement: Additional file 1 — Long term recovery of Nrg-GFP after photobleaching in epithelial cells of live embryos. Face-on views of epithelial cells in wild-type embryos. Pre-bleach image with the bleached area indicated with box. Note the partial recovery of fluorescence even until 41 min after bleaching. Bar: 5 μm. [file 1471-2121-9-38-S1.tiff]

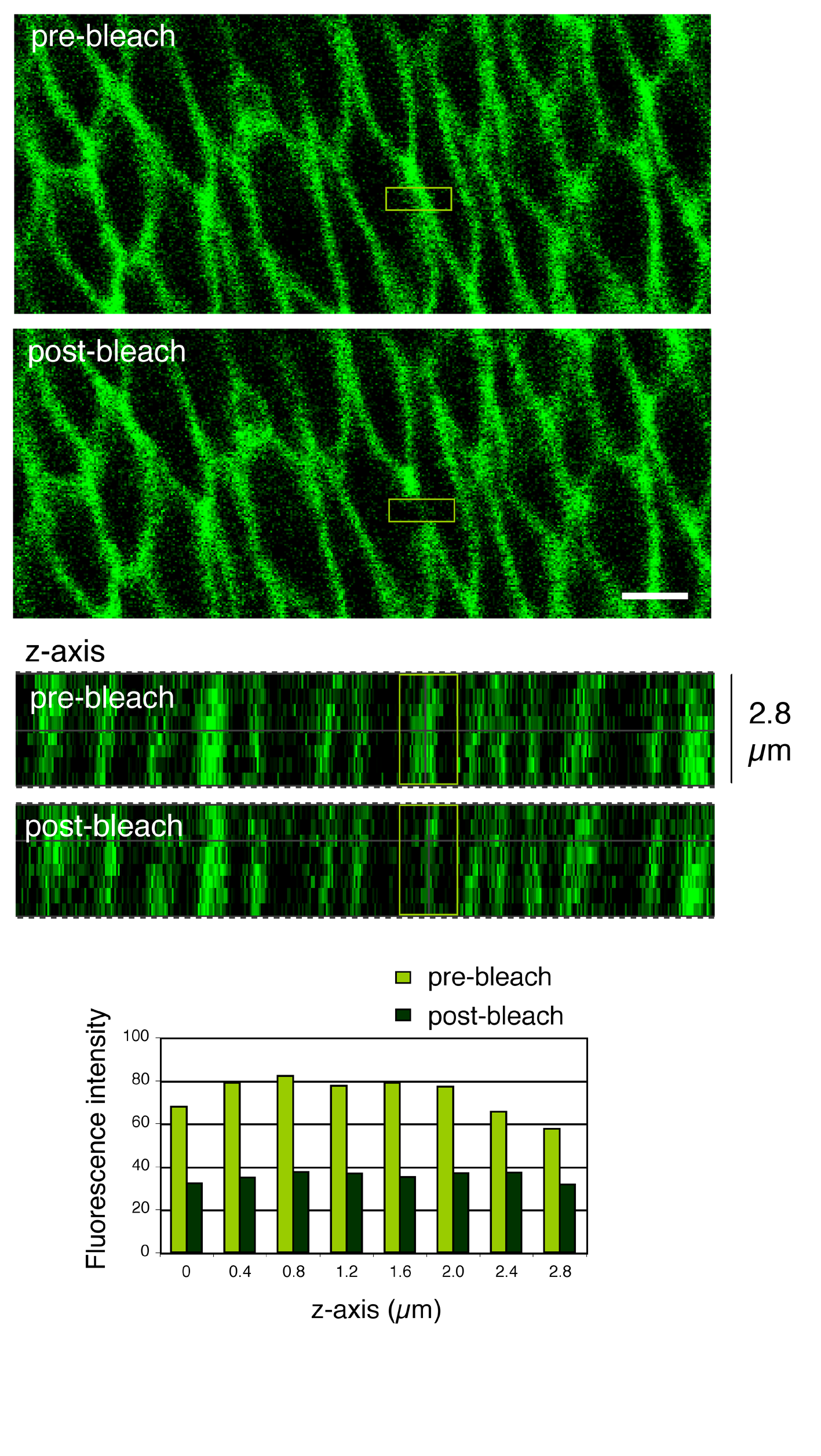

Supplement: Additional file 2 — Bleaching of Nrx IV-GFP along the z-axis in epithelial cells of live embryos. Confocal xy- and z-sections (stacks of 8 sections, 400 nm step) of epithelial cells in wild-type embryos. Pre-bleach and post-bleach images with the bleached area indicated with box. Quantification of fluorescence intensities into the bleached area of confocal sections. Bar: 5 μm. [file 1471-2121-9-38-S2.tiff]
